# Successful retrieval of migrated stent via endoscopic ultrasound-guided hepaticogastrostomy with fine-gauge dilation catheter

**DOI:** 10.1055/a-2610-2931

**Published:** 2025-06-26

**Authors:** Kohei Okamoto, Susumu Hijioka, Yoshikuni Nagashio, Shota Harai, Mark Chatto, Yutaka Saito, Takuji Okusaka

**Affiliations:** 1 68380 Department of Hepatobiliary and Pancreatic Oncology, National Cancer Center Hospital, Tokyo, Japan; 2 Department of Medicine, Makati Medical Center, Manila, Philippines; 3 68380 Endoscopy Division, National Cancer Center Hospital, Tokyo, Japan

Endoscopic ultrasound-guided hepaticogastrostomy (EUS-HGS) has been widely performed; however, serious adverse events occasionally occur. For example, intraoperative or postoperative stent migration occurs in 2.68% of cases 1 . Retrieval by endoscopic approaches is often challenging. In this report, we present a case in which a completely migrated plastic stent (PS) into the intrahepatic bile duct (IBD) through EUS-HGS was safely retrieved endoscopically using a fine-gauge biliary dilation catheter ( Video 1 ).

Successful retrieval of migrated stent via endoscopic ultrasound-guided hepaticogastrostomy with fine-gauge dilation catheter.Video 1

A 56-year-old man with advanced pancreatic cancer presented with obstructive jaundice and underwent EUS-HGS using a 7 Fr PS ( Fig. 1 ). Two weeks later, he developed cholangitis due to stent dysfunction, requiring reintervention via the HGS route. To access the incomplete fistula, a side hole was created in the PS, and a guidewire (GW) was advanced into the IBD 2 ( Fig. 2 ). Subsequently, we attempted PS removal using alligator forceps, although the stent ruptured, leading to complete migration ( Fig. 3 ). Thus, a fine-gauge biliary dilation catheter (4-mm REN; Kaneka) was inserted into the migrated stent, and intraluminal balloon inflation allowed it to be firmly grasped. The PS was easily removed, and a new metallic stent was successfully placed ( Fig. 4 ). Postoperatively, cholangitis improved rapidly, and the patient was discharged without any adverse events.

**Fig. 1:**
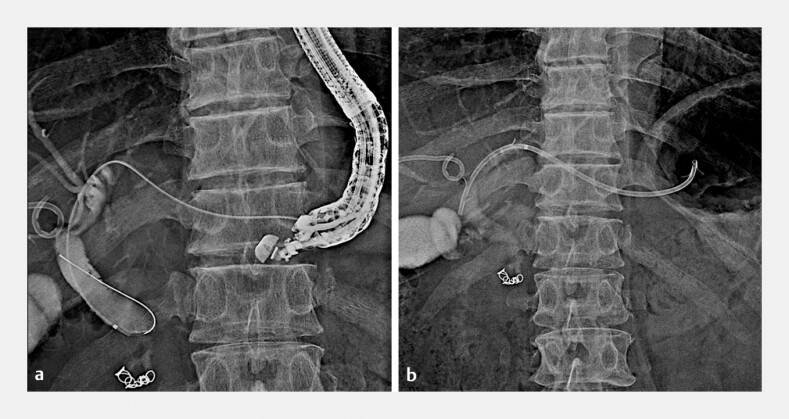
Fluoroscopic images. **a** Endoscopic ultrasound-guided hepaticogastrostomy was performed for obstructive jaundice secondary to pancreatic head cancer. **b** We punctured the B3 site and inserted a 7 Fr plastic stent.

**Fig. 2:**
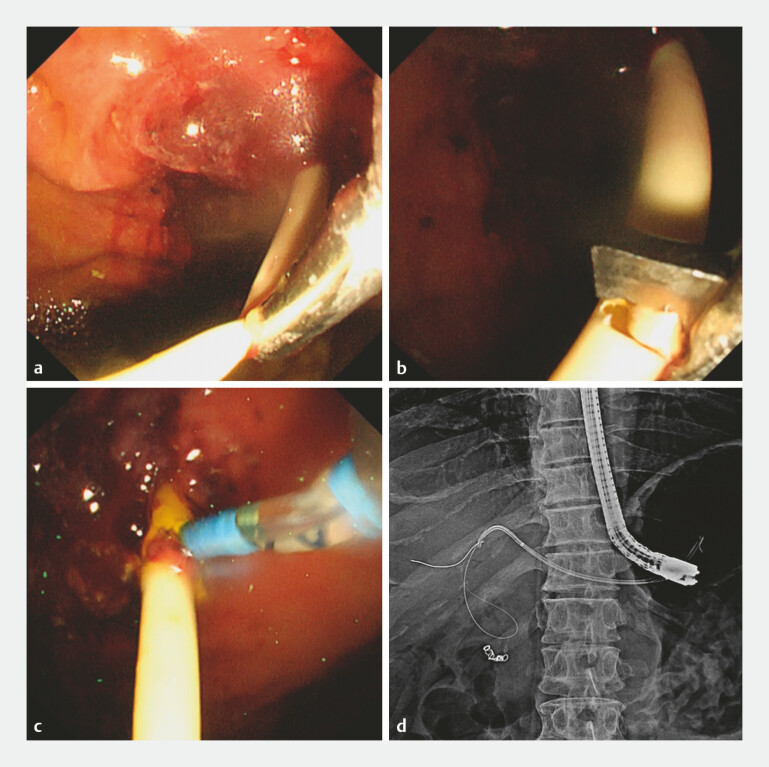
Endoscopic and fluoroscopic images. **a** Two weeks later, acute cholangitis occurred due to stent dysfunction. We attempted a stent exchange. **b–d** We cut through half of the plastic stent using a loop cutter to create a new side hole, and a guidewire was inserted.

**Fig. 3:**
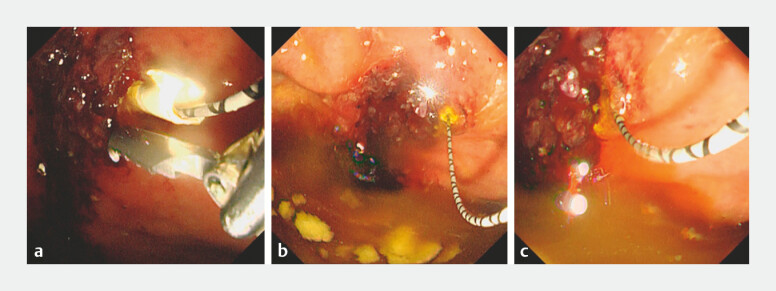
Stent migration. **a** We attempted to remove the plastic stent using alligator forceps, which was difficult due to the large fluctuations in the endoscopic field of view caused by the patientʼs deep breathing. **b, c** Finally, the plastic stent migrated into the intrahepatic bile duct via the EUS-HGS. Abbreviation: EUS-HGS, endoscopic ultrasound-guided hepaticogastrostomy.

**Fig. 4:**
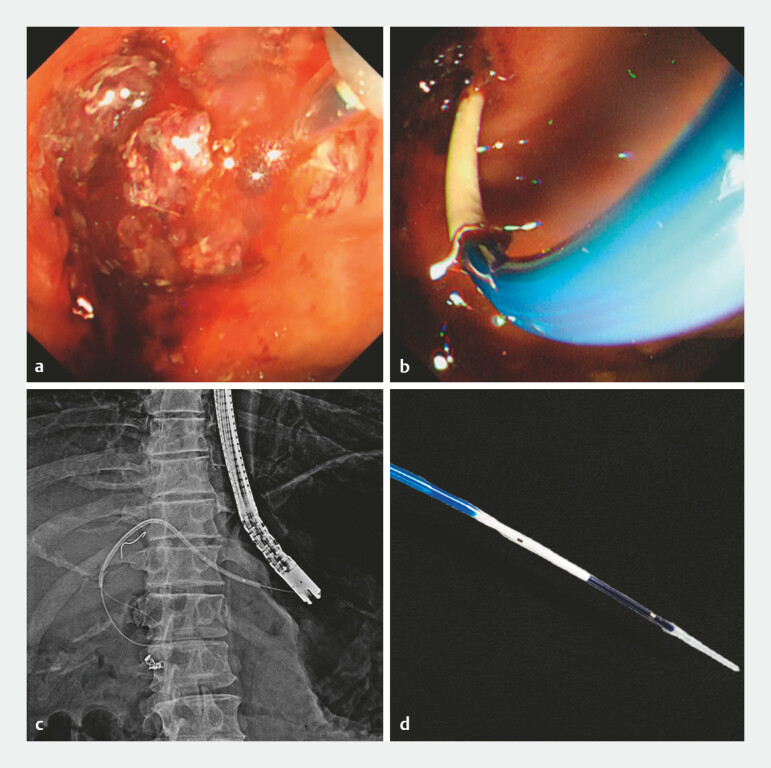
Migrated stent retrieval using a fine-gauge biliary dilation catheter. **a–c** We inserted a biliary dilation catheter into the migrated stent via EUS-HGS, dilated inside, and successfully removed the stent-balloon complex. **d** Biliary dilation catheter (4-mm, REN; KANEKA), with 2.7 Fr well-tapered distal ends for easy insertion into the plastic stent. Abbreviation: EUS-HGS, endoscopic ultrasound-guided hepaticogastrostomy.

Several methods have been reported for stent retrieval, including grasping with forceps, snares, and balloon dilation near the stent 3 4 5 . In this report, we used a biliary dilation catheter with a tapering tip, thin enough (2.7 Fr) for smooth insertion into a 7 Fr PS. The PS was removed as a complex with the catheter over the GW and through the endoscope, to minimize strain on the fistula and reduce the risk of GW deviation.

This is the first report of successful and safe retrieval of a migrated stent from the EUS-HGS route using this technique.

Endoscopy_UCTN_Code_TTT_1AS_2AH

## Notes

Endoscopy E-Videos https://eref.thieme.de/e-videos
*E-Videos* is an open access online section of the journal *Endoscopy* , reporting on interesting cases and new techniques in gastroenterological endoscopy. All papers include a high-quality video and are published with a Creative Commons CC-BY license. Endoscopy E-Videos qualify for HINARI discounts and waivers and eligibility is automatically checked during the submission process. We grant 100% waivers to articles whose corresponding authors are based in Group A countries and 50% waivers to those who are based in Group B countries as classified by Research4Life (see: https://www.research4life.org/access/eligibility/ ). This section has its own submission website at https://mc.manuscriptcentral.com/e-videos .
